# Ultra-Fast Data-Mining Hardware Architecture Based on Stochastic Computing

**DOI:** 10.1371/journal.pone.0124176

**Published:** 2015-05-08

**Authors:** Antoni Morro, Vincent Canals, Antoni Oliver, Miquel L. Alomar, Josep L. Rossello

**Affiliations:** Electronic Engineering Group, Physics Department, Universitat de les Illes Balears, Palma de Mallorca, Balears, Spain; Swiss Institute of Bioinformatics, SWITZERLAND

## Abstract

Minimal hardware implementations able to cope with the processing of large amounts of data in reasonable times are highly desired in our information-driven society. In this work we review the application of stochastic computing to probabilistic-based pattern-recognition analysis of huge database sets. The proposed technique consists in the hardware implementation of a parallel architecture implementing a similarity search of data with respect to different pre-stored categories. We design pulse-based stochastic-logic blocks to obtain an efficient pattern recognition system. The proposed architecture speeds up the screening process of huge databases by a factor of 7 when compared to a conventional digital implementation using the same hardware area.

## Introduction

Data explosion is the capability of current technologies to generate large amounts of data at different scientific disciplines. Data volumes are doubling every year in most areas of modern science [1], and its proper analysis is becoming more and more complex. As a matter of fact, data explosion has not led to an information explosion since current data analysis techniques are unable to handle billions of data records in a reasonable period of time. Large scientific databases containing several terabytes of information need to be continuously screened by scientists, and current processor-based techniques are unable to provide an efficient response to this problem. To solve this, different solutions have been developed based on artificial neural networks [2–5], the use of simple metrics [6] or the extraction of simplified datasets from the original data [7].

An alternative to traditional deterministic computational methodologies is the use of stochastic logic, introduced more than 40 years ago [8,9]. Stochastic computing is the result of applying probabilistic laws to logic cells where variables are represented by random pulse streams, thus providing a natural way of representing analog quantities with digital systems [10]. Pulses can be converted to binary numbers by using digital counters (P2B converters) while binary numbers can be translated to stochastic signals by combining a random (or a pseudo-random) number generator and a comparator (B2P converters). Stochastic computing makes use of digital technology to perform complex arithmetic operations with a reduced number of gates. When performing those types of operations, stochastic signals must be completely uncorrelated for a proper behavior.

Stochastic computing is useful for those applications requiring parallel-processing techniques [11–14]. Traditional parallel processing architectures have the shortcoming of requiring a large amount of hardware resources. Therefore, the number of tasks that can be executed in parallel within a single chip is relatively small. Stochastic computing could represent a solution to this problem since the hardware used to solve each task is reduced in size if compared to traditional digital implementations. As a result, more complex tasks can be executed in parallel when using stochastic computing elements. The greatest advantage of stochastic computing is the potential of implementing hundreds of smart computing elements in one single integrated circuit, thus obtaining a highly parallelized processing chip with a computing capacity that can be several orders of magnitude higher than traditional binary-logic-based microprocessors.

In this work we generalize the basic stochastic computing principles, thus creating a more general *probabilistic processing*. The primary basic principle of traditional stochastic computing is that the stochastic bit streams must be completely uncorrelated in time and space to obtain the desired arithmetic operations when using simple logic gates. Our extended *probabilistic processing* approach will also use correlated signals in order to implement a set of non-linear operations. This set of non-linear operations can be used for an efficient and fast comparison between signals as we will show later.

Accordingly, *probabilistic computing* is characterized by the combination of two different types of operations:

- Arithmetic operations implemented by uncorrelated signals (such as the multiplication, the division or the addition). These ones are taken from the traditional stochastic computing concepts [8].
- Non-linear operations performed by correlated signals evaluated through logic gates.

The introduction of the possibility of coherence between the signal phases enhances the capacity of stochastic computing by including those non-linear operations that can be applied to perform fast similarity searches. Consequently, complex pattern recognition tasks can be executed in parallel when using probabilistic processing operations.

In this work we present a highly efficient methodology for data mining based on probabilistic processing. High dimensional data is inherently complex in clustering, classification and similarity search [15]. The proposed approach is evaluated showing its application to a similarity search over a huge database. Most data mining algorithms use similarity search as a subroutine core [16–18], and thus the time taken for this task is the bottleneck of virtually all data mining algorithms [19]. Similarity search plays a fundamental role in many data mining and machine learning problems, e.g. text categorization [20], collaborative filtering [21], time-series analysis [22,23], protein sequencing [24] or any application-specific task as petroglyphs comparison [25]. At the same time, the mining of huge datasets implies the use of large computer clusters [26,27]. The proposed approach based on the use of probabilistic processing shows large improvements in terms of hardware resources when compared with conventional solutions.

## Basic Principles of Probabilistic Processing

### Stochastic computing principles

**Fig 1:**
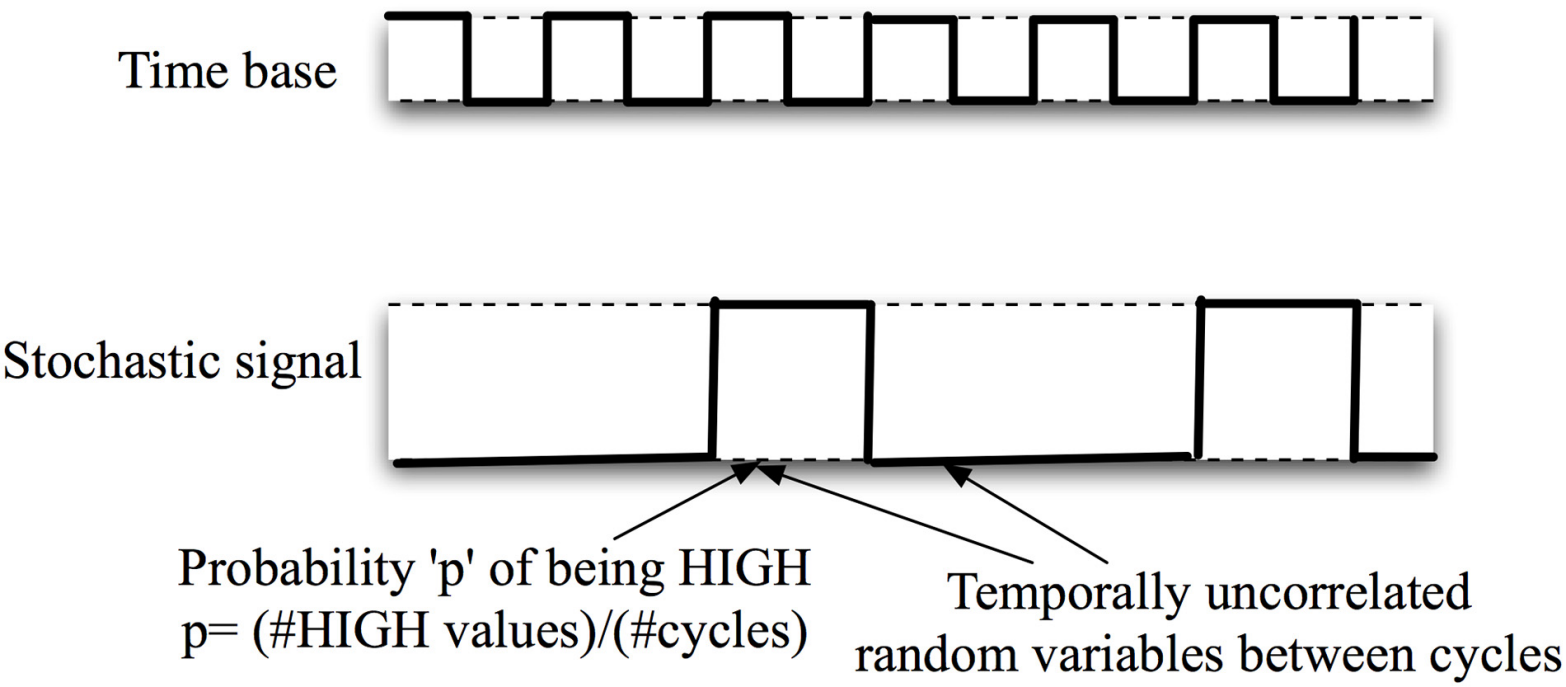
Basic temporal behavior of stochastic signals. There is no correlation between the signal values at different clock cycles. The number associated with the stochastic signal is the activation probability.

**Fig 2:**
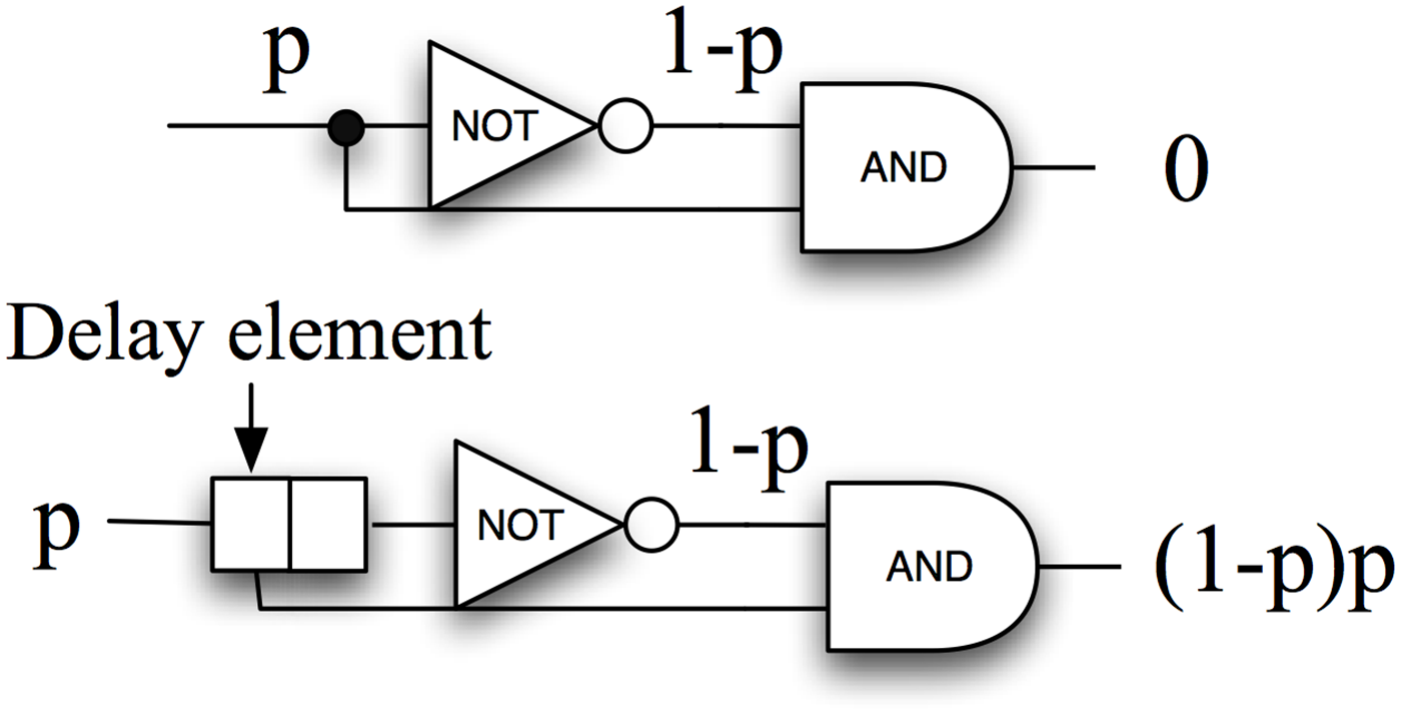
Correlation between signals and mathematical relationship between inputs and outputs. If we desire to implement the function p(1-p) we must add a delaying element to de-correlate signals at the input of the AND gate.

In stochastic-based computations a global clock provides the time interval during which all stochastic signals are stable (settled to 0 or 1). For each clock cycle, a particular node has a probability *p* of being in the HIGH state (see Fig 1). Stochastic pulsed signals follow probabilistic laws when evaluated with logic gates. As an example, an AND gate provides at the output the product of its inputs (that is to say, the collision probability between signals) whereas a NOT gate converts a probability *p* at the input to the complementary *1-p* at the output. One of the requirements for these stochastic computing blocks is that signals must be un-correlated at different clock cycles and between them. In Fig 2 we show the importance of the temporal de-correlation when implementing arithmetic functions (we use the example of implementing *f(p) = p(1-p)*). The figure illustrates that if the inputs of the AND gate *p* and *1-p* are correlated the output is always equal to zero. Such correlations can be eliminated using shift registers to delay signals from one arithmetic level to the next one. In the correct case, the AND gate evaluates properly the product between *p* and a delayed (and therefore uncorrelated) value of *1-p*.

### Random number generation

**Fig 3:**
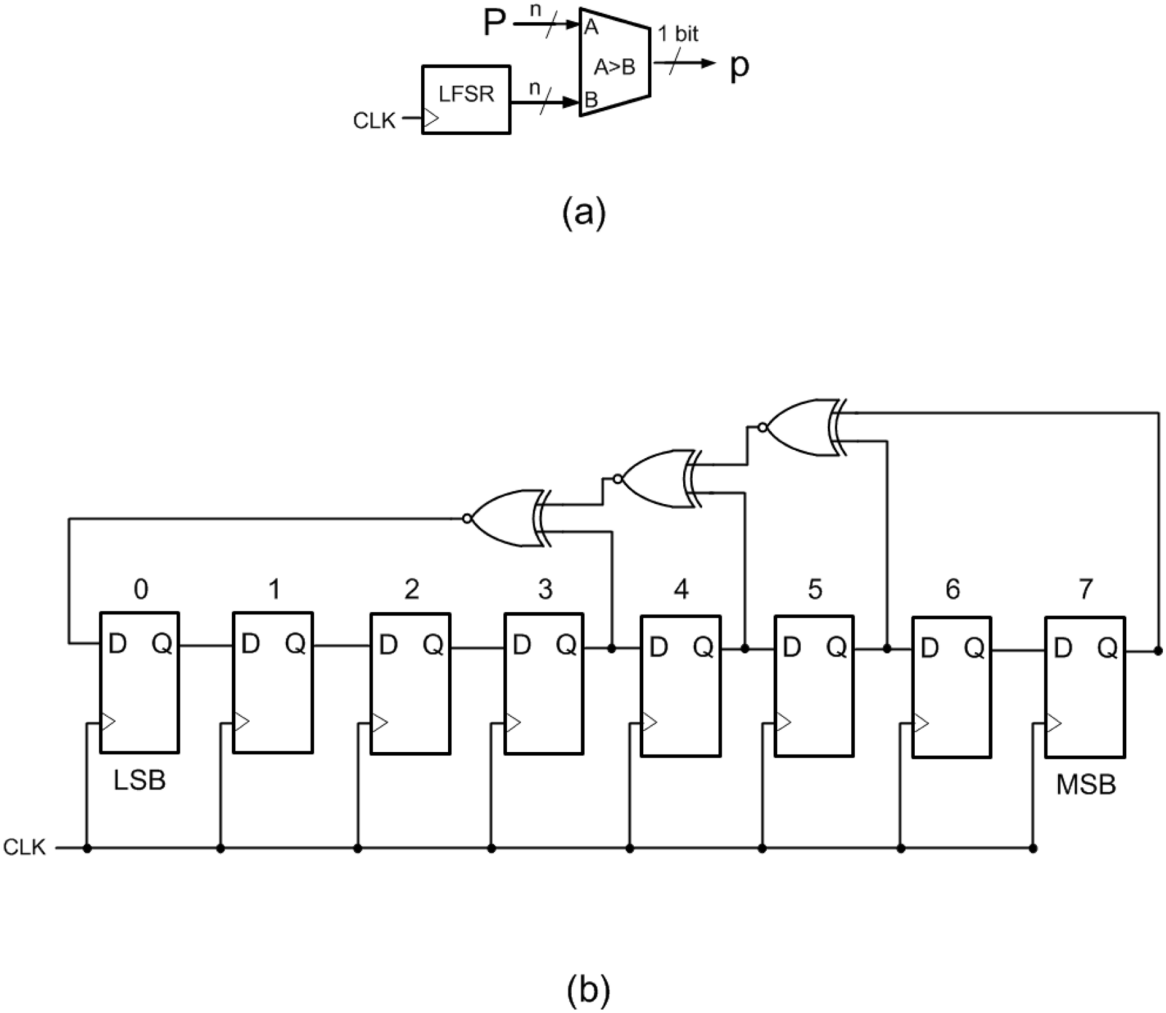
(a) Binary to pulse converter (B2P), an LFSR and a comparator are combined to obtain the pulsed signal. (b) Linear Feedback Shift Register (LFSR) used in the experiments.

The generation of pseudorandom sequences is a key issue for the implementation of probabilistic computing systems since stochastic bit streams are required to convert binary magnitudes to their equivalent stochastic signals. In particular, in order to obtain a random variable from Bernoulli sequences with a known generating probability *p*, we have to compare the binary number to convert (*P*) and the generated random number (*R*). If *P>R*, the stochastic pulse will be at high level, otherwise it will be low (Fig 3a).

**Fig 4:**
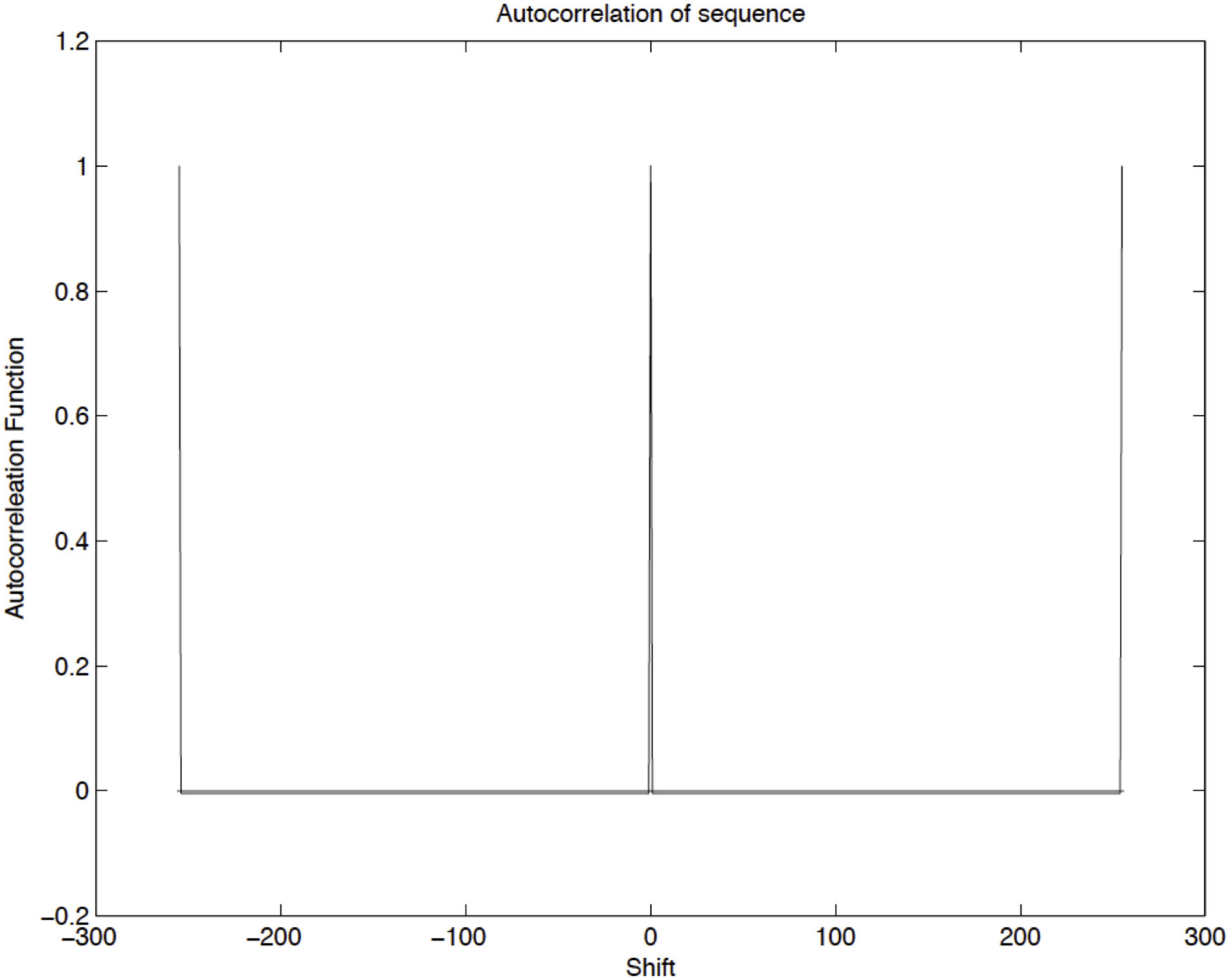
Autocorrelation function. Autocorrelation function of the pseudo-random number generator used in the experiments.

A commonly used [28] source of pseudorandom digital noise is the linear feedback shift register (LFSR), which is an array of interconnected flip-flops with feedback to its input from a combination of the outputs of its various stages gated together in EXCLUSIVE-OR gates (see Fig 3b). This linear feedback structure provides uniformly distributed sequences (which have an autocorrelation delta function), but with a finite period of repetition, which has an exponential dependence with the number of bits. Since the sequences are produced deterministically, uncorrelated sources must be generated using different initial values (seeds) for the registers. The feedback configurations enabling maximal-length generators have been given [29,30]. The configuration applied in the present work, which employs a 8-bit shift register, is shown in Fig 3b. The autocorrelation function of a pseudorandom sequence generated by the LFSR used is shown at Fig 4. This function is a set of repeating delta functions separated by the number of bits in the LFSR’s sequence length (2^n^-1).

For operations that need un-correlated signals, we define different seeds for each LFSR block. On the other hand, for operations requiring correlated signals we employ the same LFSR output for all stochastic variables.

### Generalization of stochastic computing

**Fig 5:**
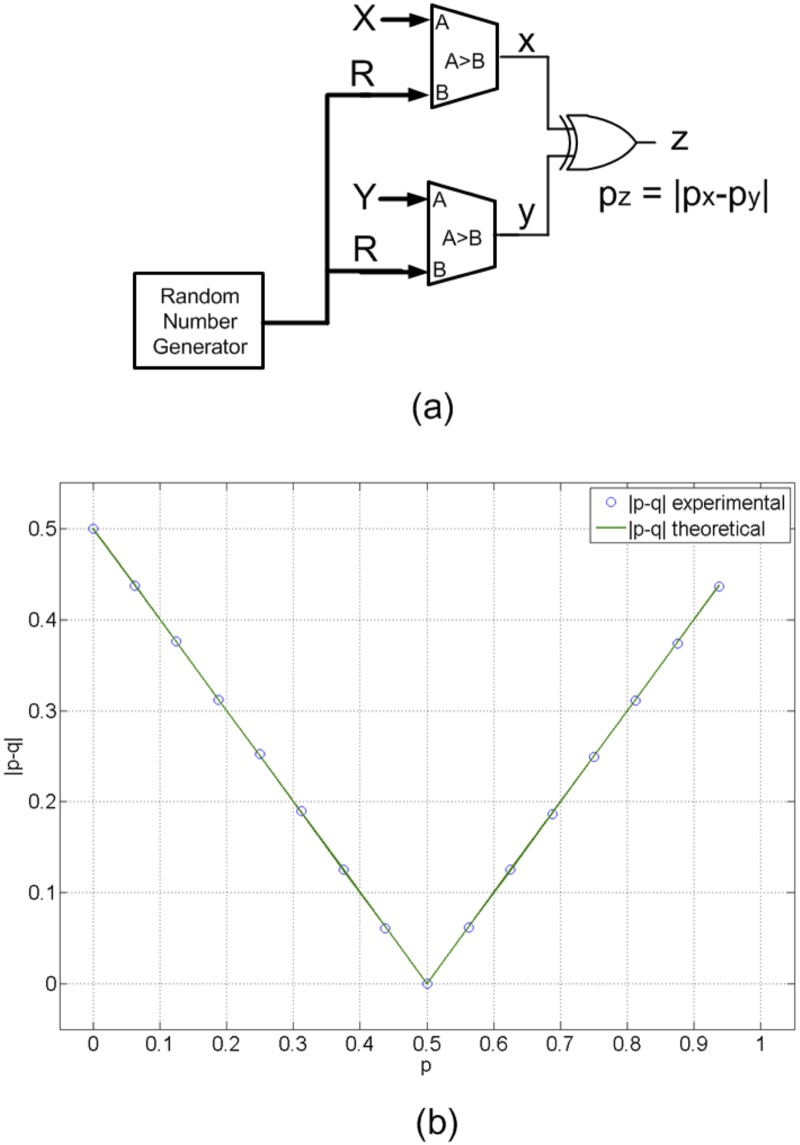
Similarity estimation of X and Y (with correlated signals). In contrast to the un-correlated scheme (case in which X and Y are compared with different random values), a non-linear function is obtained. The probability of the R signal of being between X and Y is proportional to |P_x_-P_y_| that is the switching activity of the output signal. (a) Probabilistic circuitry for the computation of |P_x_-P_y_|. (b) Experimental results when evaluating |P_x_-P_y_|.

Different kinds of stochastic non-linear functions can be reproduced using stochastic correlated signals. In Fig 5a, we show an example of the implementation of the absolute value of a subtraction (|p_x_-p_y_|). Stochastic signals x and y are derived from binary numbers X and Y when comparing with a random number R, generated using one LFSR. The probability of getting x = '1' or y = '1' (values of p_x_ and p_y_) is proportional to X and Y respectively. Since X and Y are correlated (they share the same LFSR) the probability of getting both signals x and y with a high level (x = y = '1') is min(p_x_, p_y_), while the probability of getting both signals with a low level (x = y = '0') is min(1- p_x_, 1- p_y_). These two situations provide a low signal at the XOR output. On the other hand, the probability of getting different values on x and y is equal to | p_x_-p_y_|. In this situation, the XOR output (z) provides a high value with probability p_z_ = | p_x_-p_y_|.

The experimental measurements of this circuit confirm this behavior (see Fig 5b). In general, correlated signals evaluated through logic gates would implement max-min algebra functions. These types of functionalities are ideal for pattern recognition.

### Data mining of huge databases

The probabilistic nature of stochastic logic is an advantage for the implementation of probabilistic-based pattern recognition methodologies [2] and pattern matching is in the core of many data mining processes. The purpose is to compare parameters (the features) from different objects with reference vectors that represent different categories. All the features define each object, thus configuring an m-dimensional vector (for m different features).

**Fig 6:**
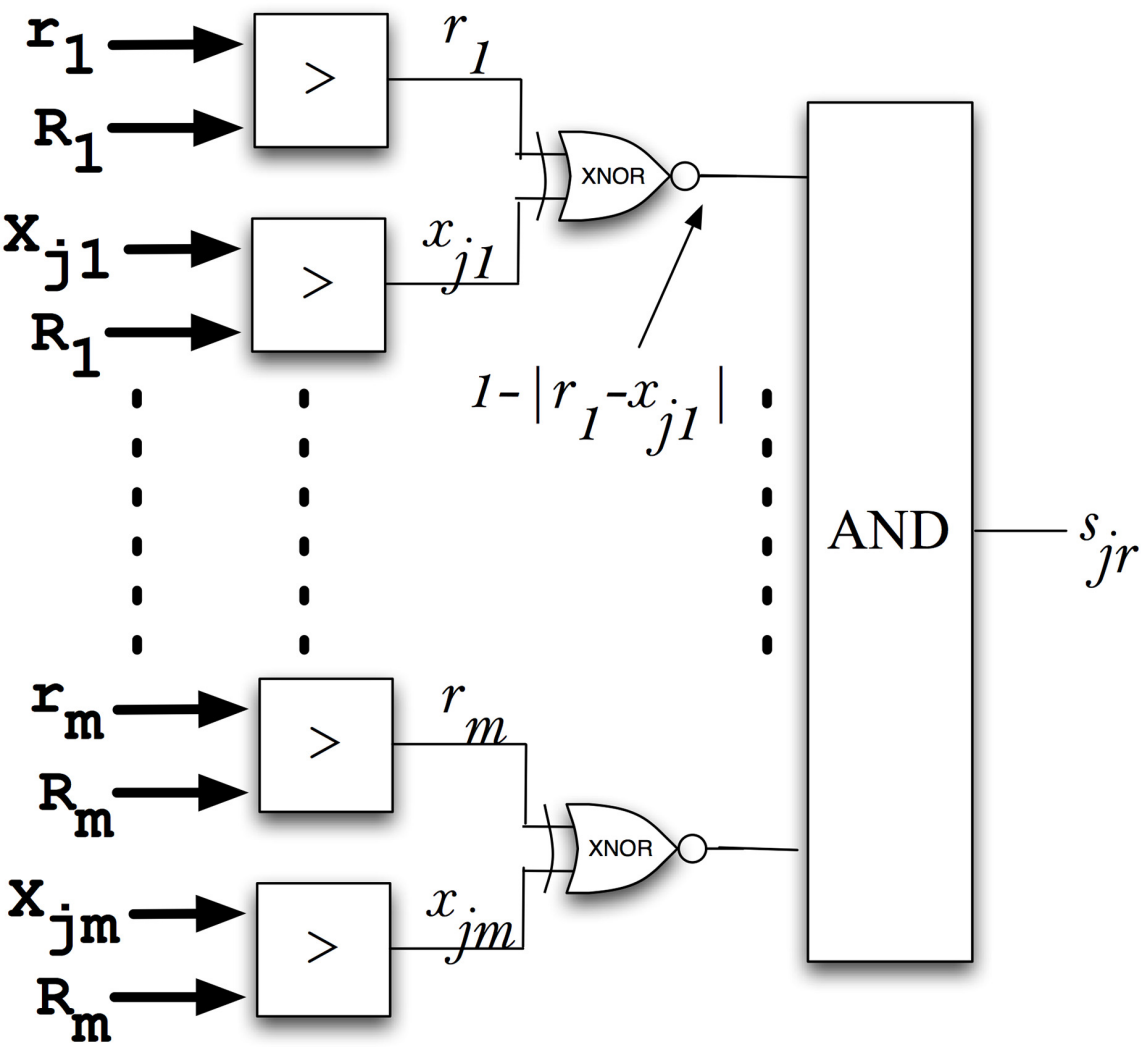
Stochastic architecture for comparing m-dimensional vectors. A total of 'm' random signals are needed for each comparison.

Fig 6 shows the stochastic circuit used to compare two m-dimensional vectors, providing at the output an estimation of the similarity (*s*_*jr*_) of both objects (vector *'* ***r*** *'* in the database to mine, and the vector defining the category '*j' (* ***x***_***j***_ *)*). A total of *'2m'* binary comparators and also *'m'* randomly selected binary numbers (*R*_*k*_ for the k-th descriptor) are configured in parallel to create a total of *2m* switching bits. Each pair of stochastic bits (*x*_*jk*_ and *r*_*k*_) are compared through XNOR gates connected with a block performing the AND function between the *m* signals, thus providing at its output an estimation of the similarity (*s*_*jr*_) between the vector (**r**) and the category j (**x**_**j**_).

Note the combination of both correlated and uncorrelated stochastic signals to obtain *s*_*jr*_ (all the AND inputs must be uncorrelated between them so that we need *'m'* random numbers *R*_*k*_). This block would represent the typical implementation of a probabilistic processing unit combining both types of signaling. Therefore, the probabilistic signal obtained (*s*_*jr*_) can be expressed as:

$$s_{jr}\;=\;\prod_{k\;=\;1}^{m}\left(1-\left|x_{jk}-r_{k}\right|\right)$$ (1)

**Fig 7:**
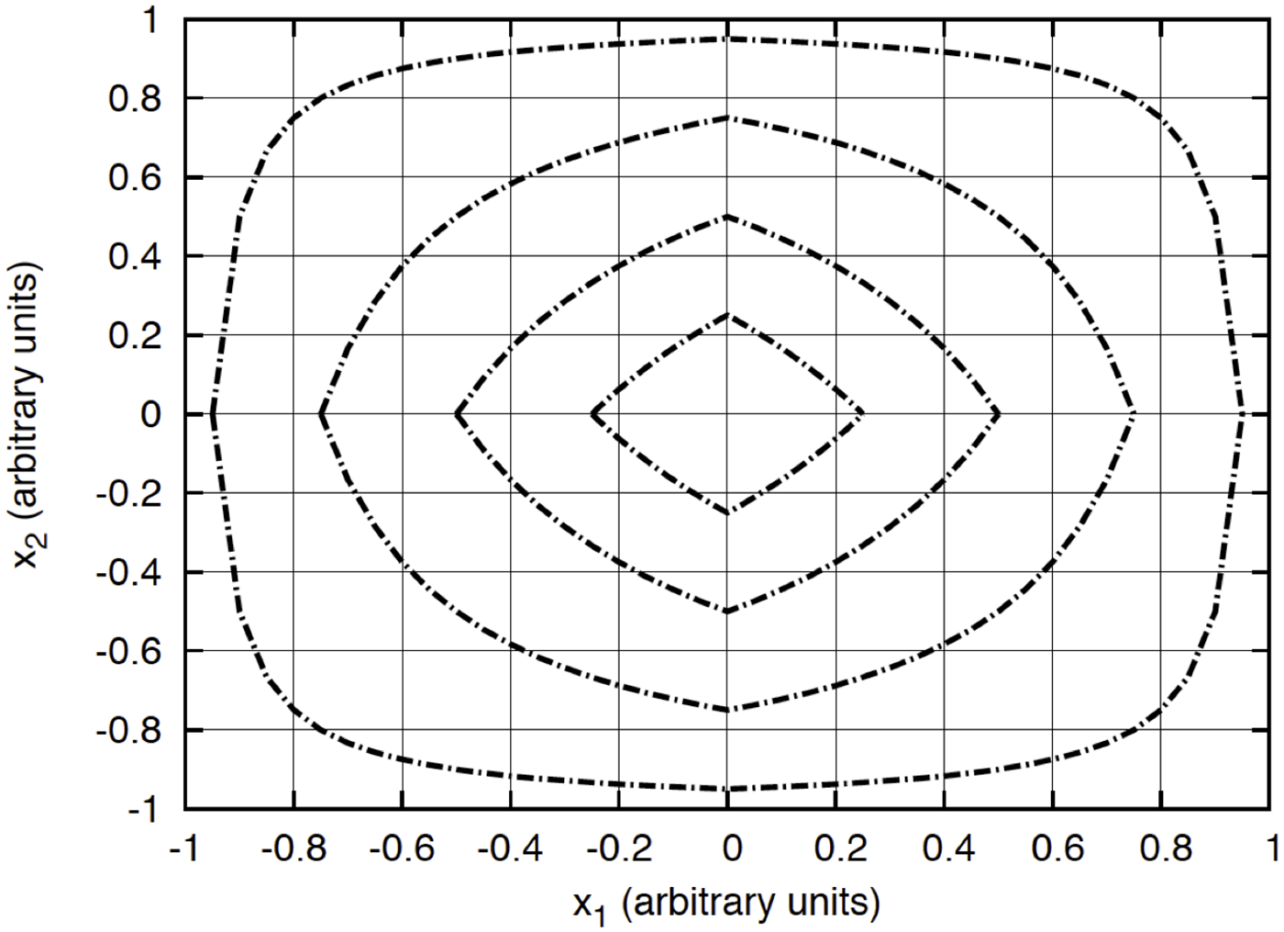
Level curves for the similarity metric used for the case of a two-dimensional space.

The switching activity at the output of the AND block is therefore proportional to the similarity between the object **r** and the category represented by vector **x**_**j**_. In Fig 7 we show the level curves associated to the Manhattan-based metric used in (1). As it is shown, the selected metric can be considered a good proximity estimator for fast mining of huge databases.

**Fig 8:**
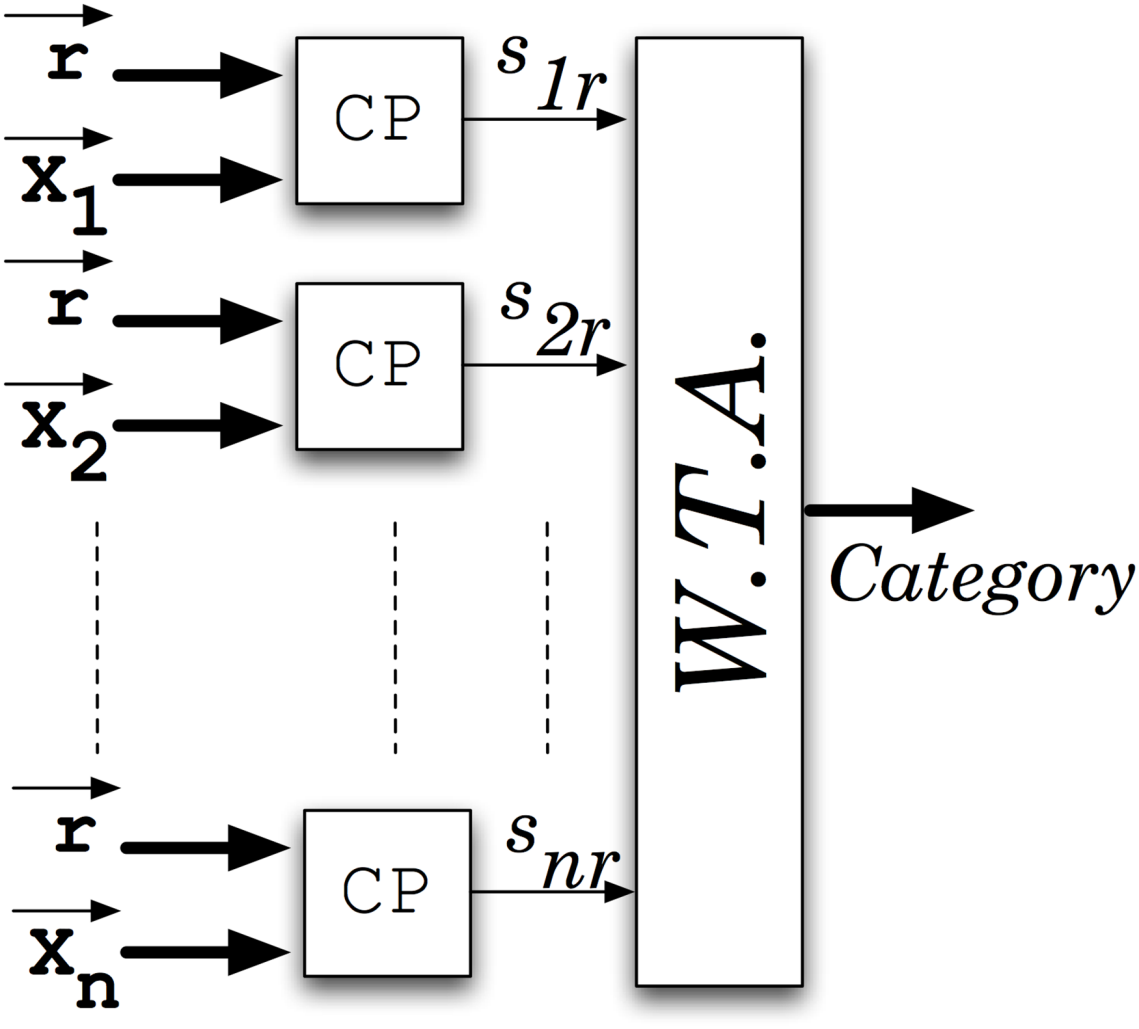
Architecture used to estimate the closest category to the reference vector 'r'. Each comparator (CP) provides at its output a switching signal proportional to the similarity of the two vectors connected to it. The Winner-Take All selects the highest frequency signal.

Hundreds of similarity estimators as the one shown in Fig 6 can be configured in parallel in a medium-sized FPGA, thus increasing considerably the mining speed in comparison with traditional processor-based techniques. Different vectors can be compared in parallel by using a Winner-Take All (WTA) architecture (see Fig 8). From '*n'* different vectors of the database, the circuitry provides at its output the closer to reference vector **r**. Then, relative fraction of the area used by the LFSRs with respect to the total circuit area decreases with the number of vectors **x**_**j**_ that can be compared.

Thus, at the WTA output the category label *'j'* that is activated is the one with the highest similarity:

$$Category\;=\;{'j'\;|\;s_{jr}\geq s}_{ir}\;\forall i\;\in \left\{1..n\right\}$$ (2)

**Fig 9:**
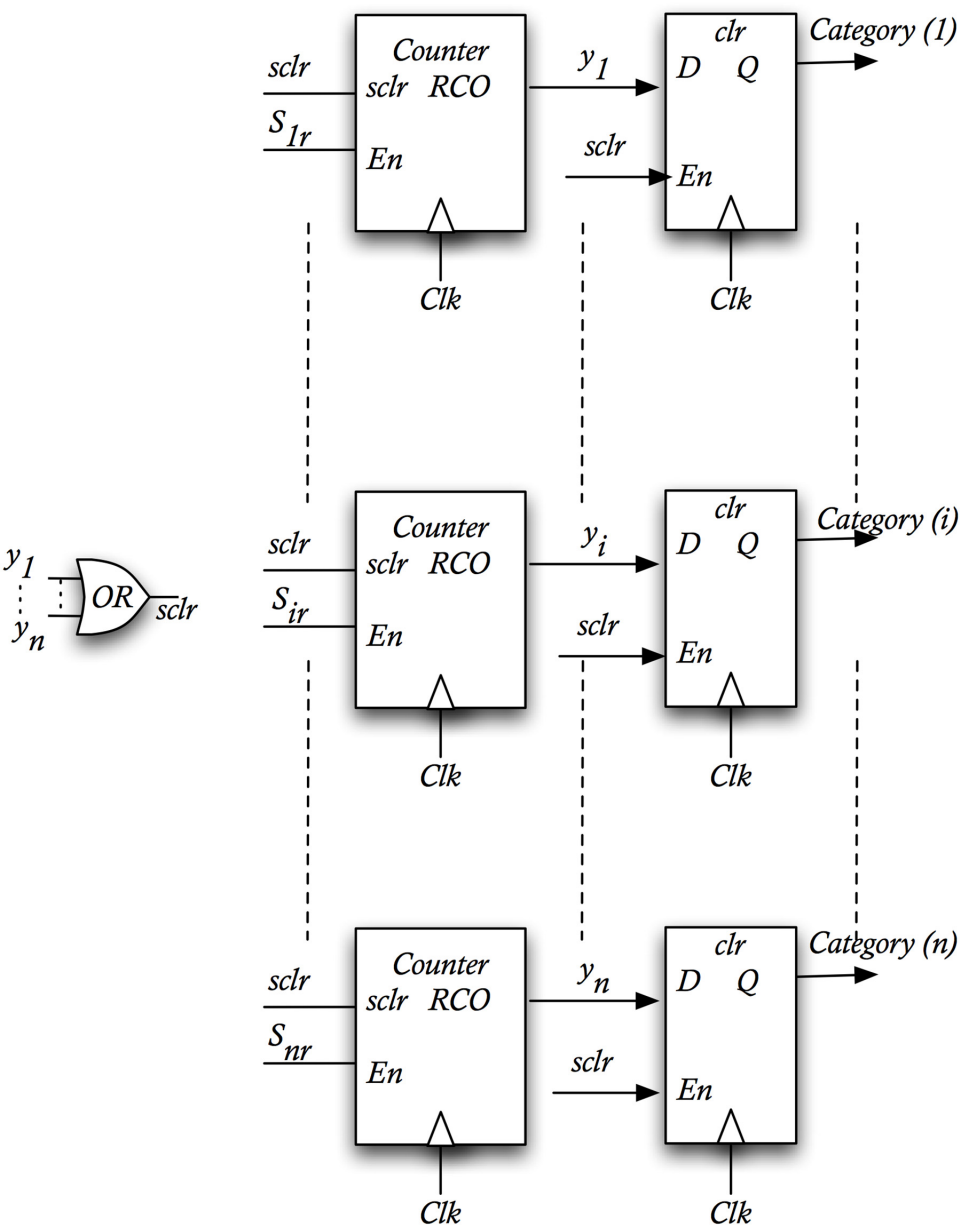
Winner-Take All. Architecture used to select the category associated to the *s*_*ir*_ with the higher activity.

The WTA can be constructed by using binary counters (module-k) (see Fig 9). Only a maximum number of clock cycles per comparison (*N>k*) are allowed, and therefore the number of cycles needed to overflow fixes a minimum similarity value to be distinguished *s*_*min*_ so that *N•s*_*min*_ = *k*. If all the similarities at the input of the WTA (*s*_*jr*_) are lower than *s*_*min*_, the most probable scenario is not to obtain any positive result at the output of the WTA.

**Fig 10:**
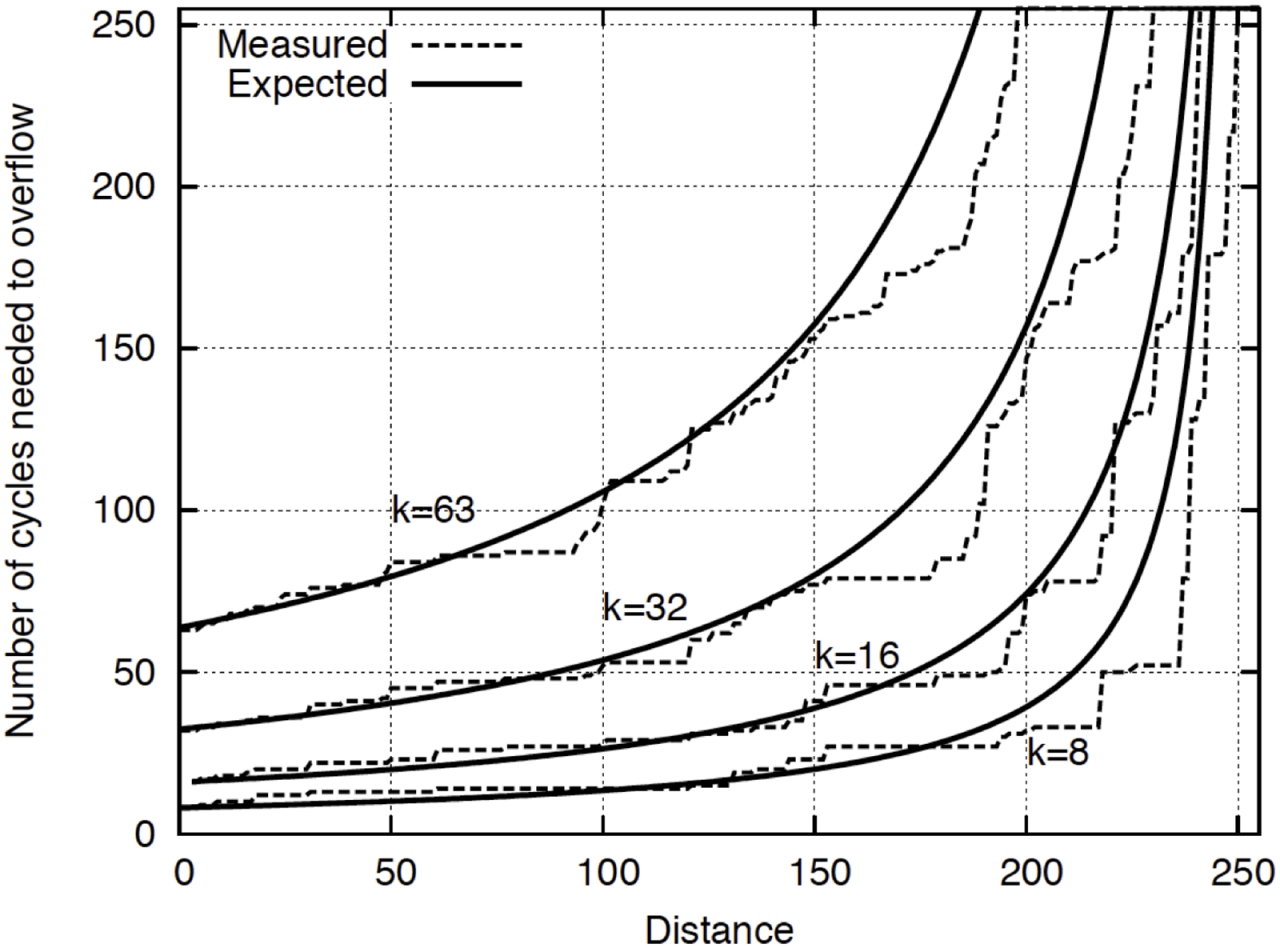
Relationship of distance between vectors (|*x-y*|) and the number of cycles needed to overflow a module-k counter of the WTA. The switching activity of the similarity stochastic signal (*s*_*jr*_) is dependent on the distance (*s = 1-d*). As the higher is the distance, lower will be the switching activity of *s*, and higher the number of cycles needed to overflow the counter.

For any similarity value *s*_*jr*_ *>s*_*min*_ between two vectors (**x**_**j**_ and **r**) we have that the probability of identifying vector ***r*** as belonging to class *'j'* is close to 1. In Fig 10 we show the relationship between the distances of two vectors (|*x-y*|) and the number of cycles (N) needed to obtain an overflow of one of the counters of the WTA. We compare the analytical formula of the theoretical behavior (solid line showing the formula *N = k/s*, where the similarity is s = 1-d since we only vary the distance in one dimension) and the measurements obtained with an ALTERA Cyclone III FPGA (dashed lines). As it can be seen, a close relationship is obtained between the expected behavior and the probabilistic implementation.

**Fig 11:**
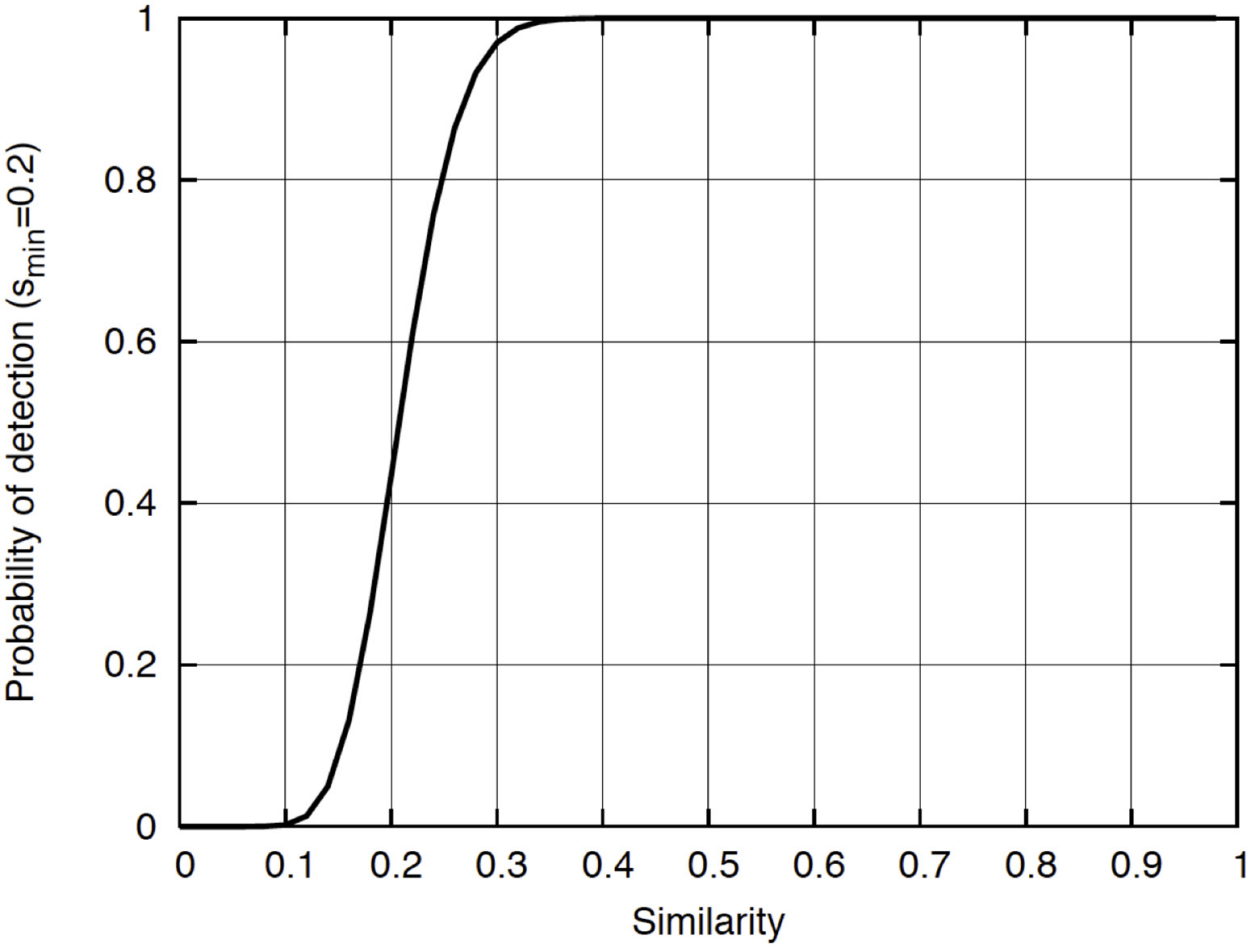
Variation of query identification probability with respect to similarity. An arbitrary threshold of *s*_*min*_ = *0*.*2* is selected.

The probability of identifying vector ***r*** as belonging to class *'j'* can be estimated considering the probability of obtaining more than *'k'* HIGH values from a signal with switching activity *'s*_*jr*_ *'* when waiting a total of N cycles (N>k).

$$P_{jr}\left(s_{jr}\right)\;=\;P(r\in \;j)\;=\;\sum_{l\;=\;k}^{N}\left(\begin{array}{c} N\\ l \end{array}\right)s_{jr}^{l}{\left(1-s_{jr}\right)}^{N-l}$$ (3)

where *N* is the total number of cycles used to compare any set of vectors. Function (3) is represented in Fig 11 for the special case in which *k = 16* and *N = 80* as a function of the similarity *s*_*jr*_. For this case, *s*_*min*_ = *k/N* = *0*.*2*, note that *P*_*jr*_ *>0*.*5* for *s*_*jr*_ *>s*_*min*_ in Fig 11.

The similarity search function (1) has the inherent advantage of its analytical simplicity. In this sense, given two randomly selected *m*-dimensional vectors **x**_**j**_ and **r**, it is possible to compute the cumulative distribution function of obtaining a similarity lower than a given selected value *'z'*. From basic probability theory we have that this cumulative distribution function can be estimated as:

$$F_{z}\left(z\right)\;=\;\int_{\Omega }^{}dy$$ (4)

where *F*_*z*_ *(z)* is the probability of obtaining a similarity lower than *z*, Ω is the volume for which *g*(***y***)<*z*, being *g(* ***y*** *)* the function under consideration (1). Vector ***y*** is composed by all the parameters for which *'g'* is dependent (the components of both **x**_**j**_ and **r** vectors that are stochastic signals with values between 0 and 1).

After some algebra, and considering (1) for the estimation of Ω and that each parameter is bounded between 0 and 1 we have:

$$F_{z}(z)\;=\;z^{2}\sum_{j\;=\;1}^{m}\frac{{\left[-2log\left(z\right)\right]}^{j-1}}{\left(j-1\right)!}\;=\;\frac{\Gamma \left(m,-2log(z)\right)}{\Gamma \left(m\right)}$$ (5)

where *'m'* is the dimension of the vectors. If we want to estimate the probability density function *f*_*z*_ *(z)*, we have to derive the cumulative distribution function with respect the selected similarity (*z)*, *f*_*z*_(*z*) = *∂*_*z*_ *F*_*z*_(*z*)

From expression (5) we can estimate the number of positive identifications from a database with *W* unknown objects if we select a minimum similarity of *s*_*min*_ (*Positives≈W•(1-F(s*_*min*_ *))*). Expression (5) is also very useful to estimate the minimum database width *W*_*min*_ needed to identify an object with a specific property (assuming a random database):

$$W_{min}\;=\;\frac{1}{1-s_{min}^{2}\sum_{j\;=\;1}^{m}\frac{{\left[-2log\left(s_{min}\right)\right]}^{j-1}}{\left(j-1\right)!}}$$ (6)

## Results

**Fig 12:**
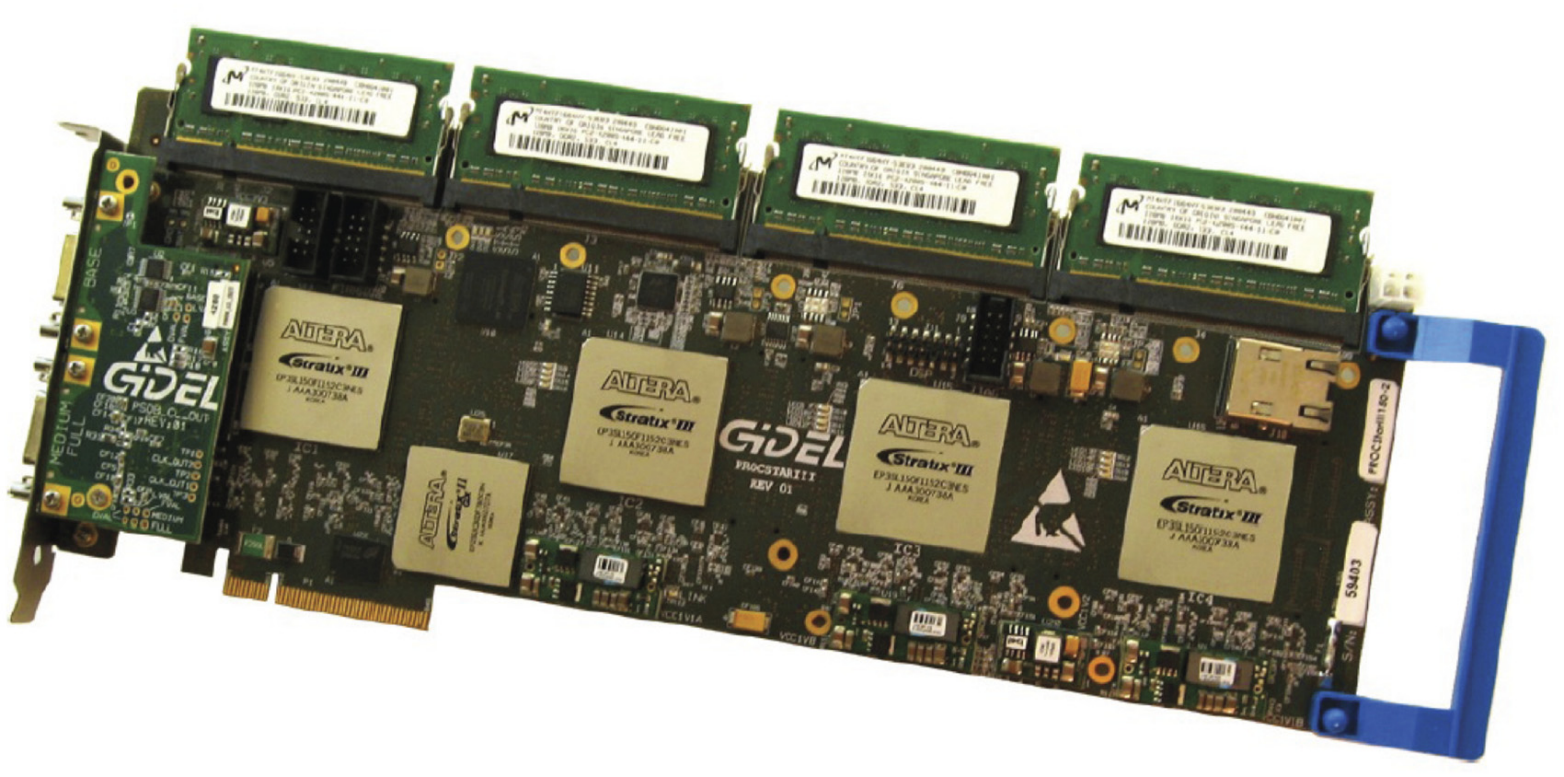
FPGA-based PCIe board used for the data mining process from GIDEL.

We implemented the proposed methodology in a FPGA-based PCIe board (ProcStarIV110E-4B) fabricated by GIDEL Ltd (see Fig 12). This board incorporates four ALTERA STRATIX III 110E FPGAs along with 32GB of DDR2 SODIMM memory banks to allocate the database. The communication with the board is done through a PCI-express connector of a PC. The clock frequency of operation of the board is 87.5 MHz and the maximum power dissipation is about 100W.

**Fig 13:**
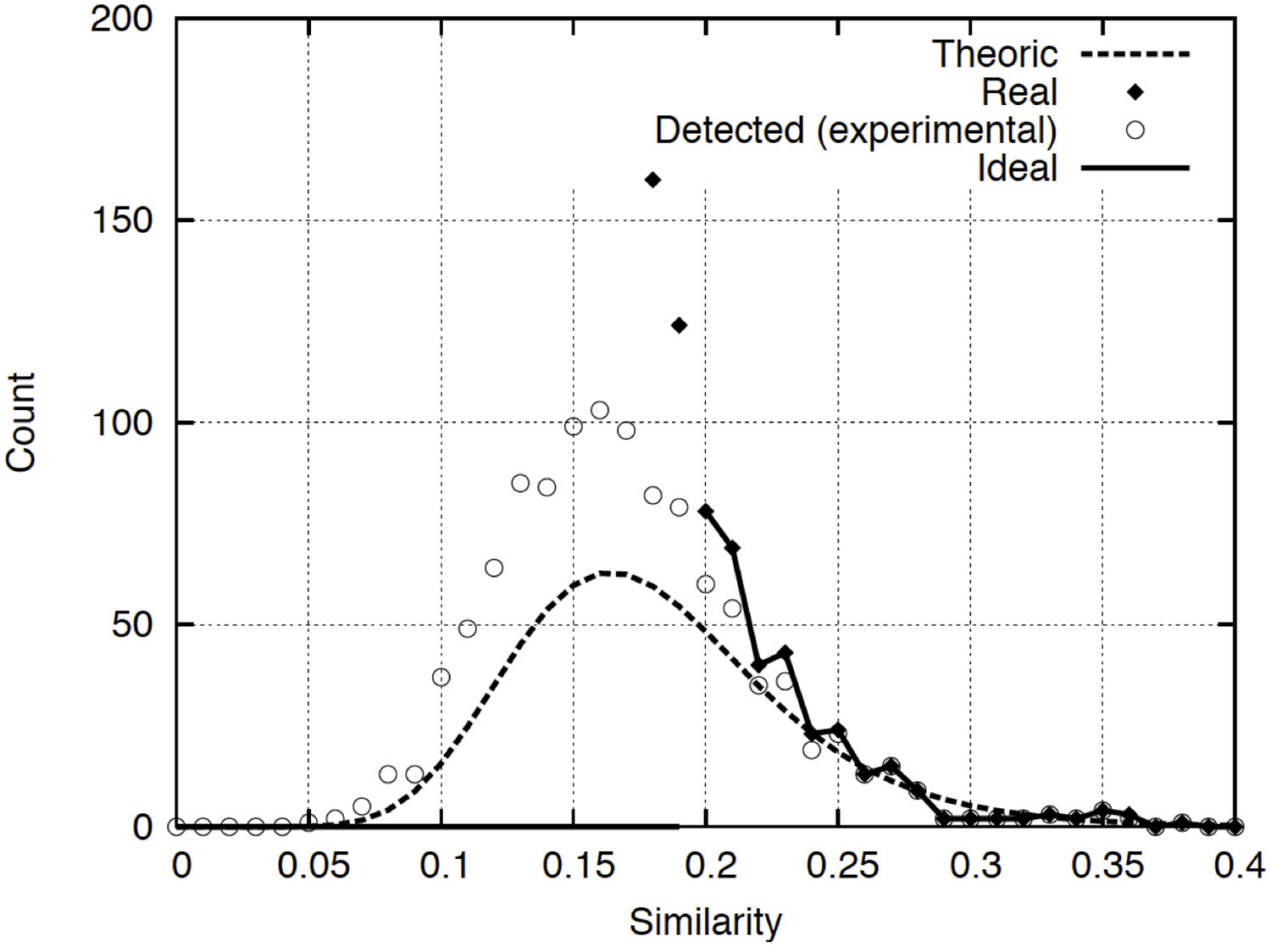
Number of positive identifications from a database with 2.56*•*10^6^ particles when *s*_*min*_ = 0.2.

Inside the four FPGA cores we configured a total of 400 similarity comparators per core (design shown in Fig 6), thus implementing a total of 1600 stochastic comparators in the board that operate in parallel. The dimension of the vectors are selected to be *m = 12*. To test the circuit effectiveness, we created a similarity search in a random database with a total of 2.56*•*10^6^ 12-D vectors setting an arbitrary minimum similarity of *s*_*min*_ = *0*.*2*. The final positive results are shown in Fig 13, where we show, as a function of the similarity '*s*': the number of vectors of the database with this similarity (dark diamonds), the number of vectors that are finally selected by the circuitry as belonging to class '*c*' (circles), the ideal number of vectors to be selected (solid line) and the expected number of vectors to be selected (dashed line *f*_*z*_(*s*_*jr*_)*P*_*jr*_). It is visible that the experimental and theoretical results (circles and dashed line) are similar. We also can see that nearly all the vectors with a similarity higher than the selected threshold are identified (circles and solid line). Unfortunately there is a non-negligible fraction of vectors with a similarity lower than *s*_*min*_ that are selected (circles with *s*<*s*_*min*_ = *0*.*2*). This fact is due to the non-negligible tie of *P*_*jr*_ *(s*_*jr*_ *)* for *s*_*jr*_<*s*_*min*_ and the exponential dependence of *f*_*z*_ *(s*_*jr*_ *)* with *s*_*jr*_.

The solution to erase such *false positives* is to filter-out the final results using software. The impact of this filtering on the database screening timing is small since only a low volume of vectors must be recomputed (rather than the billions of vectors that the database can contain).

The total number of positives to be selected by the stochastic circuitry can be estimated numerically as:

$$Positives\;=\;\int_{0}^{1}P_{jr}\left(s\right)f_{z}\left(s\right)ds$$ (7)

**Fig 14:**
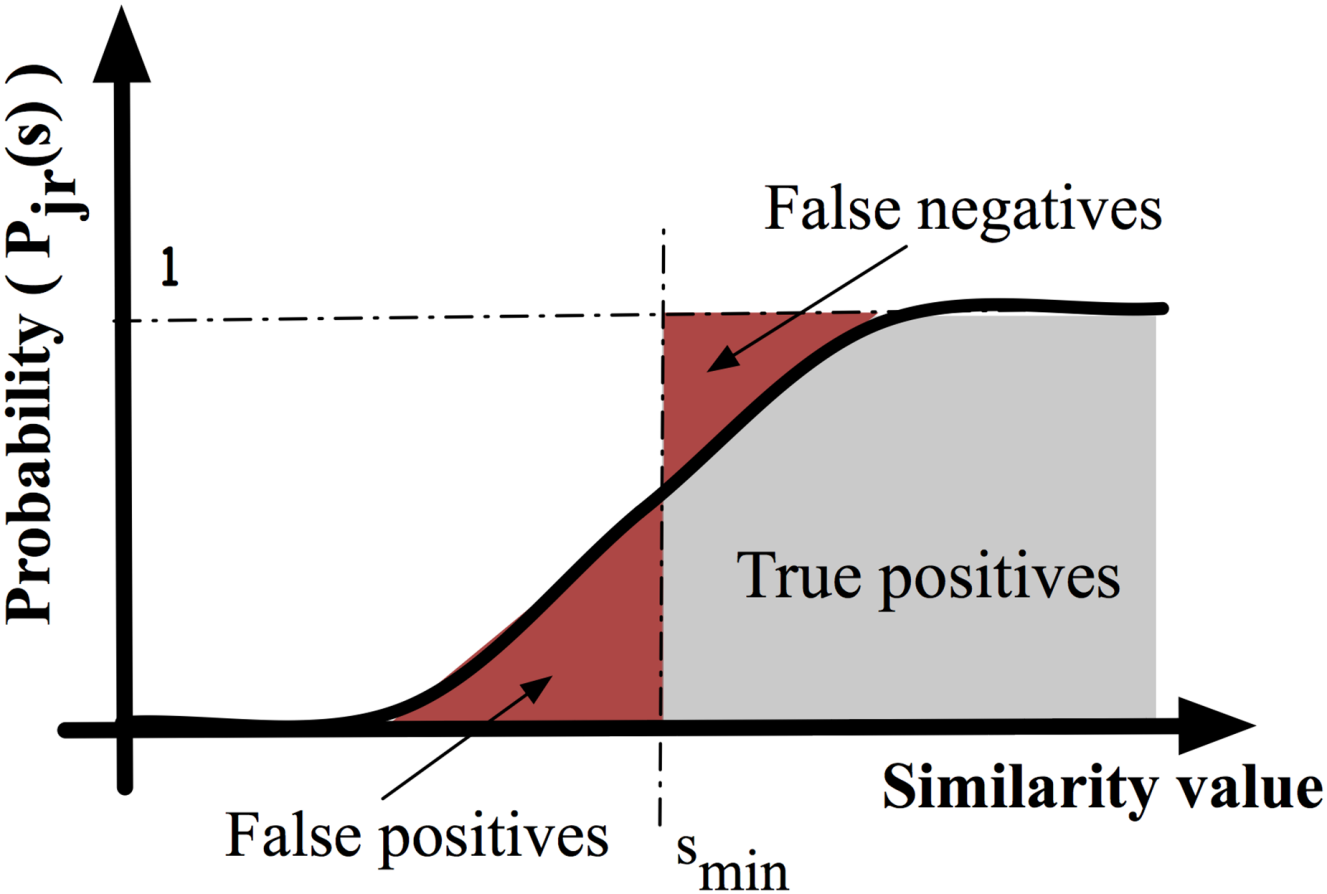
For each computation, a given number of false positives and negatives are provided by the probabilistic system. False positives can be filtered out by software while false negatives are lost.

Expression (7) can be used to select an optimal number for *k* and *N* to control the number of positive results within reasonable values. In Fig 14 we show the different types of negatives and positives provided by the system. False positives are those positives with a similarity lower than the threshold *s*_*min*_. False negatives are those vectors that, even presenting a higher similarity than *s*_*min*_, are not found by the system. Finally, the true positives and negatives are those vectors that are correctly classified by the system.

Although the false positives can be filtered-out by software, the vectors with *s>s*_*min*_ that are finally not found by the hardware (false negatives) cannot be recovered (in Fig 13, the false negatives are the difference between the solid line and the circles). In this sense, the precision of the system is defined as the ratio between true positives and the total number of possible positives (true positives + false negatives).

$$\eta \;=\;\frac{\int_{s_{min}}^{1}P_{jr}\left(s\right)f_{z}\left(s\right)ds}{\int_{s_{min}}^{1}f_{z}\left(s\right)ds}$$ (8)

In Table 1 we show the dependence of η with respect to parameter k assuming *f*_*z*_ = *1* with s_min_ = 0.5. Increasing *k* increases the efficiency but decreases the screening speed of the circuit. In Table 1 we find the relationship between η and the number of cycles needed to overflow a WTA counter (k value). For most applications, a value of k = 8 provides good results in terms of accuracy and computation time.

**Table 1:** Relationship between the k value and the precision of the system when fz = 1 and using smin = 0.5 (i.e. N = 2k).

k	Precision (η)
8	0.93
16	0.94
32	0.96
64	0.97
128	0.97

**Fig 15:**
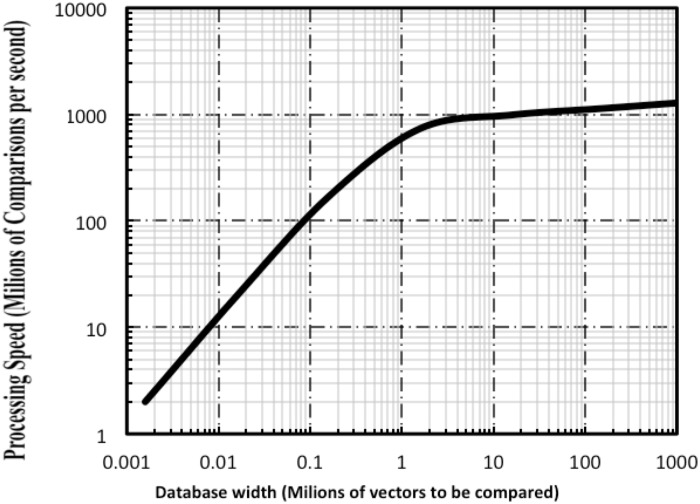
Data mining speed variation with database width when using probabilistic computing. Each comparison implies the processing of two vectors of 12 Bytes each one.

The circuit speed can be estimated from the time needed to screen a database (*t*_*p*_) with a total of *W* vectors:

$$t_{p}\;=\;\frac{W∙size}{f_{RAM}}+\frac{NT_{clk}W}{1600}+t_{setup}$$ (9)

where W is the number of vectors in the database, *f*_*RAM*_ is the data transfer frequency of the DDR-DRAM (of the order of 16GB/s for the PCIe used in the experiment), *T*_*clk*_ is the global clock period of the circuit (operating at 87.5MHz), *'size'* is the number of bytes per vector (12 bytes in our case), 1600 is the number of stochastic comparators implemented inside the FPGAs, parameter *N* is the number of clocks used by the WTA to process the inputs (signals *s*_*jr*_), and finally *t*_*setup*_ is the setup time needed for board initialization (of the order of 1ms). Finally, the speed of the screening process is estimated as a function of the number of vectors in the database (*f*_*proc*_ = *W/t*_*p*_). In Fig 15 we show the screening speed (in millions of comparisons per second) as a function of the database width (in millions of vectors), where the vectors are composed by 12 bytes (*m = 12*). The low speed obtained by the proposed methodology when the database is small is basically due to the fixed setup time of the system.

**Fig 16:**
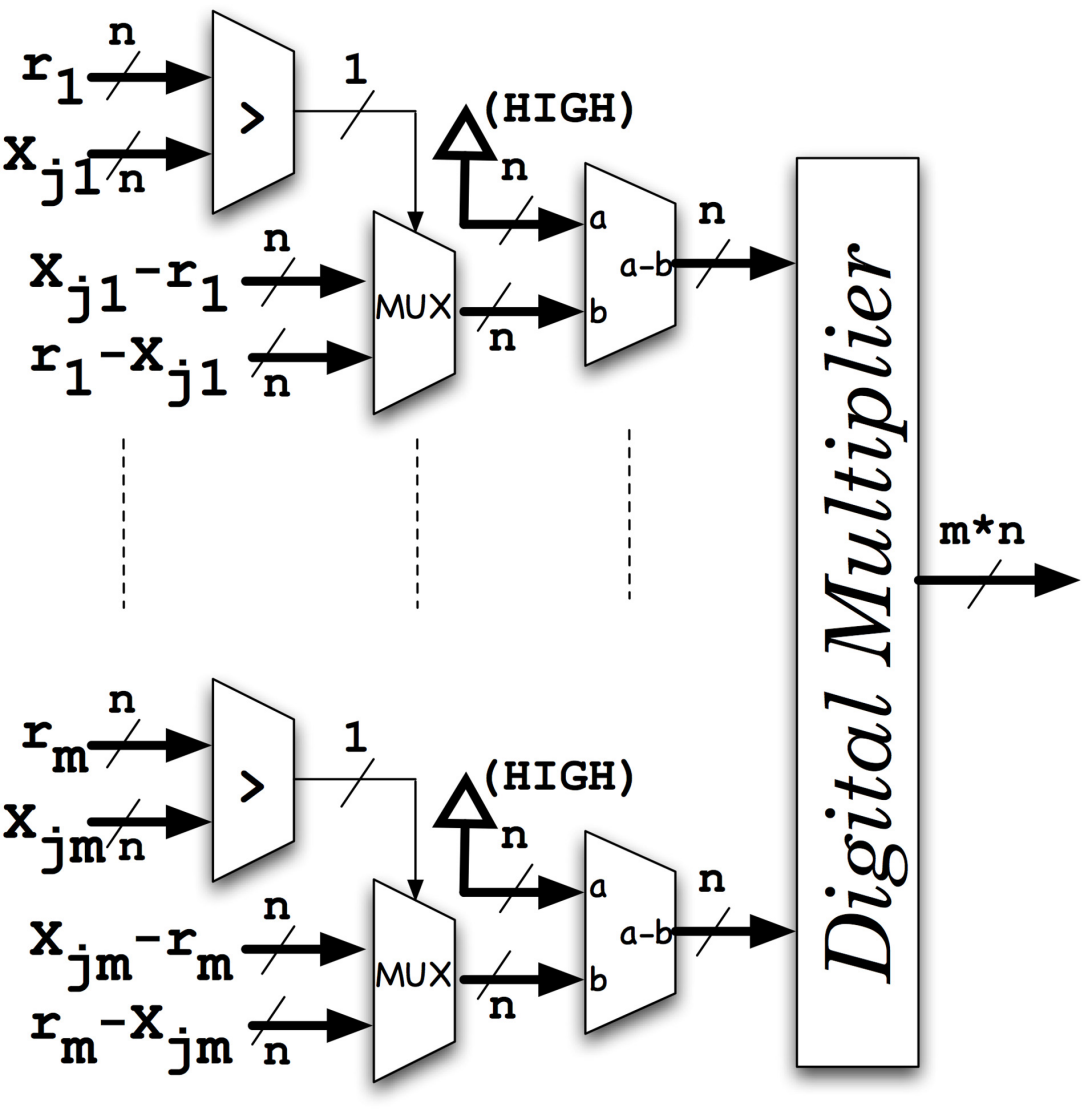
Conventional implementation of a m-dimensional digital comparator where expression (1) is implemented. The first comparator and the multiplexer implement the absolute value function while the multiplier provide the product for all the dimensions.

**Fig 17:**
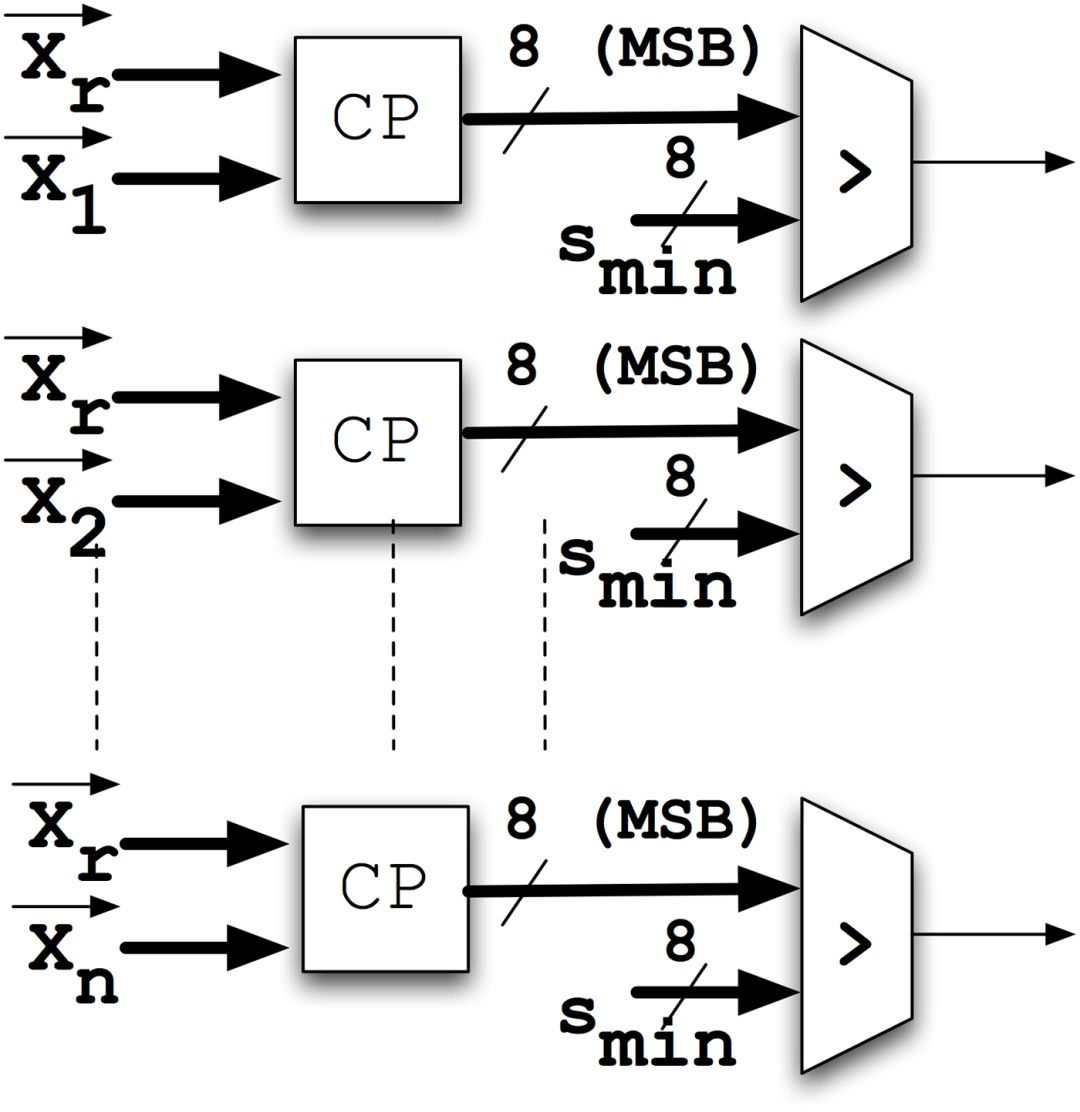
Global comparator between the reference vector and each vector in the database. Block CP is implementing the circuitry shown in Fig 16. The result is compared with *s*_*min*_.

We compared a conventional implementation using binary-based digital hardware of a multi-vector comparator implementing the metric proposed in (1) with the proposed probabilistic system. In Fig 16 we show the conventional implementation of expression (1). Finally, in Fig 17 we compare the result of such similarity comparators with the minimum similarity s_min_. In Table 2 we compare both implementations in terms of FPGA resources when using an ALTERA Cyclone III EP3C25F324C8 device. The vectors to be compared are fixed to 12 dimensions. As it can be seen, the ratio of Logic Elements needed by the conventional and the probabilistic implementation increases as the number of vectors to be compared grows. The area ratio between both systems is of the order of 56 while the ratio in terms of circuit speed is constant and about 1/k (1/8). Therefore, the probabilistic implementation speeds up the screening process of the database by a factor of 7 when compared to a conventional digital implementation using the same hardware area. In other words, the total performance when using the proposed architecture is higher by a factor of 7. It means that, the lack in speed can be compensated by using less hardware area.

**Table 2:** Relationship between conventional and probabilistic implementation in terms of FPGA resources for an Altera Cyclone III device.

Number of vectors	Classical			Probabilistic		
	Logic Elements (LE)	Multipliers	Delay time (t_D_)	Logic Elements	Multipliers	Delay time (t_D_)
2	1.955	126	1	321	0	8
4	13.898	132	1	551	0	8
8	39.142	132	1	1.023	0	8
16	91.464	132	1	1.908	0	8
32	196.076	132	1	3.713	0	8
128	823.814	132	1	14.719	0	8

To sum up, the presented application is an example where stochastic computing is advantageous over conventional computing systems. Since the presented implementation takes advantage of the high parallelism of stochastic computing, less circuit area implies a lower power demand to achieve a determined processing speed. The source code of both implementations (conventional and probabilistic) can be found in S1 Appendix.

## Conclusions

We have presented a new and unconventional computing technique for ultra-fast mining of huge databases. The methodology is based on the use of probabilistic pulsed signals. We describe how correlated bit streams can be used to implement non-linear functions like the absolute value function, which have been also developed by other research group [31]. In the final architecture we allow the use of both correlated and uncorrelated stochastic bit streams. The combination of both types of switching signals increases the mathematical capacity of original stochastic computing implementations. The data to be mined is translated to spikes and processed by a simple digital circuitry. The simplicity of the circuitry is used to implement hundreds of stochastic comparators inside Field-Programmable Gate Arrays and oriented to screen huge databases. The final implementation uses an FPGA-based PCIe board for the screening. This implementation uses less hardware resources than conventional digital methodologies (based on binary and not probabilistic logic) and is able to process the order of 13GBytes of information per second (in contrast to the estimated 2GBytes/s of speed that could be achieved by the conventional implementation using the same hardware area). With the 12-dimensional space used to allocate each vector in the example shown in this paper we obtain the order of 1 billion of comparisons per second. A patent application has been done for this new mining methodology [32].

## Supporting Information

S1 Appendix VHDL Source Codes. (PDF)
