# Supplementary material for: Ultra-Fast Data-Mining Hardware Architecture Based on Stochastic Computing
Source: PLoS One. 2015 May 8;10(5):e0124176. doi: 10.1371/journal.pone.0124176 (PMC4425430; doi:10.1371/journal.pone.0124176)
Supplement: S1 Appendix — (PDF) [file pone.0124176.s001.pdf]

Supporting Information:

S1 Appendix: VHDL Source Codes

Here we show the two approaches followed (the probabilistic and the classical implementation) in which a reference 12-D vector is compared with other two 12-D vectors. The next files compose the probabilistic implementation:

mytypes.vhd
b2pvector.vhd
lfsr_8.vhd
counter64.vhd
design3.vhd

Finally, two files compose the classical implementation in which binary logic is used:

mytypes.vhd
designclassic.vhd

VHDL Probabilistic design for a 2-vectors comparator:

```
-- Josep L. Rossello. Universitat Illes Balears. Agost 2013.

LIBRARY ieee;
USE ieee.std_logic_1164.ALL;
USE work.mytypes.all;
ENTITY design3 IS

PORT (
        clock, rst, en:         IN STD_LOGIC;
        vector   :              IN buffer_out;
        qin   :                 IN integer range 0 to 63;
        lab : OUT std_logic_vector(vectors-1 downto 0)
);

END design3;

ARCHITECTURE arch OF design3 IS

component b2pvector

        port
        (
        lfsr:       IN STD_LOGIC_VECTOR(8*parametres-1 DOWNTO 0);
        valor :     IN STD_LOGIC_VECTOR(8*parametres-1 DOWNTO 0);
        salida:     OUT std_logic_vector(parametres-1 downto 0)
        );

end component;

component lfsr_8

port

        (clk,reset,enable: IN STD_LOGIC;
        tmpo:       IN STD_LOGIC_VECTOR(8 DOWNTO 1);
        number:     OUT STD_LOGIC_VECTOR(7 DOWNTO 0)
```

```
		);

end component;

component counter64

			port(
			clk		: in std_logic;
			resetext	: in std_logic;
			reset		: in std_logic;
			enable		: in std_logic;
			qin		: in integer range 0 to 63;
			rco				: out std_logic

			);

end component;

SIGNAL lfs: STD_LOGIC_VECTOR(8*parametres-1 DOWNTO 0);
SIGNAL r: std_logic_vector(parametres-1 downto 0);
SIGNAL x1: std_logic_vector(parametres-1 downto 0);
SIGNAL x2: std_logic_vector(parametres-1 downto 0);
SIGNAL d1,rco1: STD_LOGIC;
SIGNAL d2,rco2: STD_LOGIC;
SIGNAL rstint: STD_LOGIC;



BEGIN

lfsr0: lfsr_8 PORT MAP(clock,rst,en,X"00",lfs(7 downto 0));
lfsr1: lfsr_8 PORT MAP(clock,rst,en,X"01",lfs(15 downto 8));
lfsr2: lfsr_8 PORT MAP(clock,rst,en,X"02",lfs(23 downto 16));
lfsr3: lfsr_8 PORT MAP(clock,rst,en,X"03",lfs(31 downto 24));
lfsr4: lfsr_8 PORT MAP(clock,rst,en,X"04",lfs(39 downto 32));
lfsr5: lfsr_8 PORT MAP(clock,rst,en,X"05",lfs(47 downto 40));
lfsr6: lfsr_8 PORT MAP(clock,rst,en,X"06",lfs(55 downto 48));
lfsr7: lfsr_8 PORT MAP(clock,rst,en,X"07",lfs(63 downto 56));
lfsr8: lfsr_8 PORT MAP(clock,rst,en,X"08",lfs(71 downto 64));
lfsr9: lfsr_8 PORT MAP(clock,rst,en,X"09",lfs(79 downto 72));
lfsr10: lfsr_8 PORT MAP(clock,rst,en,X"10",lfs(87 downto 80));
lfsr11: lfsr_8 PORT MAP(clock,rst,en,X"11",lfs(95 downto 88));

bloc0: b2pvector PORT MAP (lfs,vector(0),r);

bloc1: b2pvector PORT MAP (lfs,vector(1),x1);
d1 <= (r(0) XNOR x1(0)) AND (r(1) XNOR x1(1))  AND (r(2) XNOR x1(2))  AND (r(3) XNOR x1(3))  AND (r(4) XNOR x1(4))  AND (r(5) XNOR
x1(5))  AND (r(6) XNOR x1(6))  AND (r(7) XNOR x1(7))  AND (r(8) XNOR x1(8))  AND (r(9) XNOR x1(9))  AND (r(10) XNOR x1(10))  AND
(r(11) XNOR x1(11)) ;
count1: counter64 PORT MAP (clock,rst,rstint,d1,qin,rco1);

bloc2: b2pvector PORT MAP (lfs,vector(2),x2);
d2 <= (r(0) XNOR x2(0)) AND (r(1) XNOR x2(1))  AND (r(2) XNOR x2(2))  AND (r(3) XNOR x2(3))  AND (r(4) XNOR x2(4))  AND (r(5) XNOR
x2(5))  AND (r(6) XNOR x2(6))  AND (r(7) XNOR x2(7))  AND (r(8) XNOR x2(8))  AND (r(9) XNOR x2(9))  AND (r(10) XNOR x2(10))  AND
(r(11) XNOR x2(11)) ;
count2: counter64 PORT MAP (clock,rst,rstint,d2,qin,rco2);

rstint<= rco1 OR  rco2;

lab(0)<=rco1;
lab(1)<=rco2;

END arch;

------------------------------------------------------------------------
-- Josep L. Rossello. Universitat Illes Balears. Ago 2013.
-- Binary b2p

LIBRARY ieee;
USE ieee.std_logic_1164.ALL;
USE work.mytypes.all;
```

```
ENTITY B2Pvector IS

PORT (
		lfsr  : IN STD_LOGIC_VECTOR(8*parametres-1 DOWNTO 0);
		valor :	IN STD_LOGIC_VECTOR(8*parametres-1 DOWNTO 0);
		salida:	OUT std_logic_vector(parametres-1 downto 0)
);

END B2Pvector;

ARCHITECTURE arch OF B2Pvector IS

BEGIN

salida(0) <= '1' WHEN valor(7 downto 0)>lfsr(7 downto 0) else '0';
salida(1) <= '1' WHEN valor(15 downto 8)>lfsr(15 downto 8) else '0';
salida(2) <= '1' WHEN valor(23 downto 16)>lfsr(23 downto 16) else '0';
salida(3) <= '1' WHEN valor(31 downto 24)>lfsr(31 downto 24) else '0';
salida(4) <= '1' WHEN valor(39 downto 32)>lfsr(39 downto 32) else '0';
salida(5) <= '1' WHEN valor(47 downto 40)>lfsr(47 downto 40) else '0';
salida(6) <= '1' WHEN valor(55 downto 48)>lfsr(55 downto 48) else '0';
salida(7) <= '1' WHEN valor(63 downto 56)>lfsr(63 downto 56) else '0';
salida(8) <= '1' WHEN valor(71 downto 64)>lfsr(71 downto 64) else '0';
salida(9) <= '1' WHEN valor(79 downto 72)>lfsr(79 downto 72) else '0';
salida(10) <= '1' WHEN valor(87 downto 80)>lfsr(87 downto 80) else '0';
salida(11) <= '1' WHEN valor(95 downto 88)>lfsr(95 downto 88) else '0';

END arch;

--------------------------------------------------------------------------
LIBRARY ieee;
USE ieee.std_logic_1164.ALL;
--USE ieee.std_logic_arith.all;
--USE ieee.std_logic_unsigned.all;

ENTITY lfsr_8 IS
PORT (clk,reset,enable: IN STD_LOGIC;
		tmpo:	IN STD_LOGIC_VECTOR(8 DOWNTO 1);
		number:	OUT STD_LOGIC_VECTOR(7 DOWNTO 0)
);
END lfsr_8;

ARCHITECTURE arch OF lfsr_8 IS
SIGNAL tmp: STD_LOGIC_VECTOR(8 DOWNTO 1);
BEGIN
PROCESS (clk,reset)
		BEGIN
		IF reset ='1' THEN
				tmp<=tmpo;
		ELSIF (enable='1') THEN
		IF (clk'EVENT AND clk='1') THEN
				for i in 1 to 7 loop
	  tmp(i+1) <= tmp(i);
	end loop;
	tmp(1) <= tmp(8) XNOR tmp(6) XNOR tmp(5) XNOR tmp(4);
		END IF;
		END IF;
END PROCESS;
number <=tmp;
END arch;

--------------------------------------------------------------------------
-- Josep L. Rossello. Universitat Illes Balears. Oct 2011.
-- Binary Counter

library ieee;
use ieee.std_logic_1164.all;
use ieee.numeric_std.all;

entity counter64 is
		port
		(
```

```
		clk		: in std_logic;
		resetext	: in std_logic;
		reset		: in std_logic;
		enable		: in std_logic;
		qin		: in integer range 0 to 63;
		rco		: out std_logic

	);

end entity;

architecture rtl of counter64 is
signal rst: std_logic;

signal cout: std_logic;

begin

rco<= cout;
rst<= reset AND (NOT cout);
	process (clk)
	variable   cnt		: integer range 0 to 63;
	begin
		if (rising_edge(clk)) then
			if resetext = '1' then
				-- Reset extern del counter to 0
				cnt := 0;
				cout<='0';

			elsif enable = '1' then
			if rst = '1' then
				cnt := 0;
				cout<='0';
				-- Increment the counter if counting is enabled
			end if;
			if cnt=qin then
				cnt:=qin;
				cout<='1';
			elsif cnt=qin-1 then
				cout<='1';
				cnt := qin;
			else
				cnt:=cnt+1;
				cout<='0';
			end if;
			end if;
		end if;
	end process;
rco<=cout;

end rtl;
```

VHDL Classical design for a 2-vectors comparator

```
-- Josep L. Rossello. Universitat Illes Balears. Abril 2014.

LIBRARY ieee;
USE IEEE.STD_LOGIC_ARITH.ALL;
USE IEEE.STD_LOGIC_UNSIGNED.ALL;
USE ieee.std_logic_1164.ALL;
USE work.mytypes.all;

ENTITY designclassic IS
PORT (
        clock, rst, en:     IN STD_LOGIC;
        vector   :          IN buffer_out;                   -- Vectors to be compared
        qin   :             IN std_logic_vector(5 downto 0);          -- Similarity threshold
        lab : OUT std_logic_vector(vectors-1 downto 0)       -- Output results

);

END designclassic;

ARCHITECTURE arch OF designclassic IS

SIGNAL lfs: STD_LOGIC_VECTOR(8*parametres-1 DOWNTO 0);
SIGNAL r: std_logic_vector(parametres-1 downto 0);
SIGNAL c : buffer_out;
SIGNAL x : buffer_out;
SIGNAL y : comp_out;
BEGIN


c(1)(7 downto 0)<= X"FF"-vector(0)(7 downto 0)+vector(1)(7 downto 0) WHEN vector(0)(7 downto 0)>vector(1)(7 downto 0) ELSE X"FF"-vector(1)(7 downto 0)+vector(0)(7 downto 0);

c(1)(15 downto 8)<= X"FF"-vector(0)(15 downto 8)+vector(1)(15 downto 8) WHEN vector(0)(15 downto 8)>vector(1)(15 downto 8) ELSE X"FF"-vector(1)(15 downto 8)+vector(0)(15 downto 8);

c(1)(23 downto 16)<= X"FF"-vector(0)(23 downto 16)+vector(1)(23 downto 16) WHEN vector(0)(23 downto 16)>vector(1)(23 downto 16) ELSE X"FF"-vector(1)(23 downto 16)+vector(0)(23 downto 16);

c(1)(31 downto 24)<= X"FF"-vector(0)(31 downto 24)+vector(1)(31 downto 24) WHEN vector(0)(31 downto 24)>vector(1)(31 downto 24) ELSE X"FF"-vector(1)(31 downto 24)+vector(0)(31 downto 24);

c(1)(39 downto 32)<= X"FF"-vector(0)(39 downto 32)+vector(1)(39 downto 32) WHEN vector(0)(39 downto 32)>vector(1)(39 downto 32) ELSE X"FF"-vector(1)(39 downto 32)+vector(0)(39 downto 32);

c(1)(47 downto 40)<= X"FF"-vector(0)(47 downto 40)+vector(1)(47 downto 40) WHEN vector(0)(47 downto 40)>vector(1)(47 downto 40) ELSE X"FF"-vector(1)(47 downto 40)+vector(0)(47 downto 40);

c(1)(55 downto 48)<= X"FF"-vector(0)(55 downto 48)+vector(1)(55 downto 48) WHEN vector(0)(55 downto 48)>vector(1)(55 downto 48) ELSE X"FF"-vector(1)(55 downto 48)+vector(0)(55 downto 48);

c(1)(63 downto 56)<= X"FF"-vector(0)(63 downto 56)+vector(1)(63 downto 56) WHEN vector(0)(63 downto 56)>vector(1)(63 downto 56) ELSE X"FF"-vector(1)(63 downto 56)+vector(0)(63 downto 56);

c(1)(71 downto 64)<= X"FF"-vector(0)(71 downto 64)+vector(1)(71 downto 64) WHEN vector(0)(71 downto 64)>vector(1)(71 downto 64) ELSE X"FF"-vector(1)(71 downto 64)+vector(0)(71 downto 64);

c(1)(79 downto 72)<= X"FF"-vector(0)(79 downto 72)+vector(1)(79 downto 72) WHEN vector(0)(79 downto 72)>vector(1)(79 downto 72) ELSE X"FF"-vector(1)(79 downto 72)+vector(0)(79 downto 72);

c(1)(87 downto 80)<= X"FF"-vector(0)(87 downto 80)+vector(1)(87 downto 80) WHEN vector(0)(87 downto 80)>vector(1)(87 downto 80) ELSE X"FF"-vector(1)(87 downto 80)+vector(0)(87 downto 80);

c(1)(95 downto 88)<= X"FF"-vector(0)(95 downto 88)+vector(1)(95 downto 88) WHEN vector(0)(95 downto 88)>vector(1)(95 downto 88) ELSE X"FF"-vector(1)(95 downto 88)+vector(0)(95 downto 88);

c(2)(7 downto 0)<= X"FF"-vector(0)(7 downto 0)+vector(2)(7 downto 0) WHEN vector(0)(7 downto 0)>vector(2)(7 downto 0) ELSE X"FF"-vector(2)(7 downto 0)+vector(0)(7 downto 0);
```

```
c(2)(15 downto 8)<= X"FF"-vector(0)(15 downto 8)+vector(2)(15 downto 8) WHEN vector(0)(15 downto 8)>vector(2)(15 downto 8) ELSE X"FF"-vector(2)(15 downto 8)+vector(0)(15 downto 8);

c(2)(23 downto 16)<= X"FF"-vector(0)(23 downto 16)+vector(2)(23 downto 16) WHEN vector(0)(23 downto 16)>vector(2)(23 downto 16) ELSE X"FF"-vector(2)(23 downto 16)+vector(0)(23 downto 16);

c(2)(31 downto 24)<= X"FF"-vector(0)(31 downto 24)+vector(2)(31 downto 24) WHEN vector(0)(31 downto 24)>vector(2)(31 downto 24) ELSE X"FF"-vector(2)(31 downto 24)+vector(0)(31 downto 24);

c(2)(39 downto 32)<= X"FF"-vector(0)(39 downto 32)+vector(2)(39 downto 32) WHEN vector(0)(39 downto 32)>vector(2)(39 downto 32) ELSE X"FF"-vector(2)(39 downto 32)+vector(0)(39 downto 32);

c(2)(47 downto 40)<= X"FF"-vector(0)(47 downto 40)+vector(2)(47 downto 40) WHEN vector(0)(47 downto 40)>vector(2)(47 downto 40) ELSE X"FF"-vector(2)(47 downto 40)+vector(0)(47 downto 40);

c(2)(55 downto 48)<= X"FF"-vector(0)(55 downto 48)+vector(2)(55 downto 48) WHEN vector(0)(55 downto 48)>vector(2)(55 downto 48) ELSE X"FF"-vector(2)(55 downto 48)+vector(0)(55 downto 48);

c(2)(63 downto 56)<= X"FF"-vector(0)(63 downto 56)+vector(2)(63 downto 56) WHEN vector(0)(63 downto 56)>vector(2)(63 downto 56) ELSE X"FF"-vector(2)(63 downto 56)+vector(0)(63 downto 56);

c(2)(71 downto 64)<= X"FF"-vector(0)(71 downto 64)+vector(2)(71 downto 64) WHEN vector(0)(71 downto 64)>vector(2)(71 downto 64) ELSE X"FF"-vector(2)(71 downto 64)+vector(0)(71 downto 64);

c(2)(79 downto 72)<= X"FF"-vector(0)(79 downto 72)+vector(2)(79 downto 72) WHEN vector(0)(79 downto 72)>vector(2)(79 downto 72) ELSE X"FF"-vector(2)(79 downto 72)+vector(0)(79 downto 72);

c(2)(87 downto 80)<= X"FF"-vector(0)(87 downto 80)+vector(2)(87 downto 80) WHEN vector(0)(87 downto 80)>vector(2)(87 downto 80) ELSE X"FF"-vector(2)(87 downto 80)+vector(0)(87 downto 80);

c(2)(95 downto 88)<= X"FF"-vector(0)(95 downto 88)+vector(2)(95 downto 88) WHEN vector(0)(95 downto 88)>vector(2)(95 downto 88) ELSE X"FF"-vector(2)(95 downto 88)+vector(0)(95 downto 88);

x(1)(95 downto 0)<=c(1)(7 downto 0)*c(1)(15 downto 8)*c(1)(23 downto 16)*c(1)(31 downto 24)*c(1)(39 downto 32)*c(1)(47 downto 40)*c(1)(55 downto 48)*c(1)(63 downto 56)*c(1)(71 downto 64)*c(1)(79 downto 72)*c(1)(87 downto 80)*c(1)(95 downto 88);

x(2)(95 downto 0)<=c(2)(7 downto 0)*c(2)(15 downto 8)*c(2)(23 downto 16)*c(2)(31 downto 24)*c(2)(39 downto 32)*c(2)(47 downto 40)*c(2)(55 downto 48)*c(2)(63 downto 56)*c(2)(71 downto 64)*c(2)(79 downto 72)*c(2)(87 downto 80)*c(2)(95 downto 88);

lab(0)<='1' when x(1)(95 downto 90)>qin else '0';
lab(1)<='1' when x(2)(95 downto 90)>qin else '0';

END arch;
```

VHDL Common libraries

```
-- Josep L. Rossello. Universitat Illes Balears. Agost 2013.

LIBRARY ieee;
USE ieee.std_logic_1164.ALL;

package mytypes is
constant parametres: integer:=12;
constant vectors: integer:=2;

type memory_type is array (0 to 8) of std_logic_vector(31 downto 0);
type comp_out is array (0 to vectors) of std_logic_vector(vectors downto 0);type vref_type is array (0 to parametres/4-1) of std_logic_vector(31 downto 0);
type buffer_out is array (0 to 2) of std_logic_vector(95 downto 0);
type label_type is array (0 to 0) of std_logic_vector(31 downto 0);
type   arr_11x0_7x0  is array  ( 11 downto 0 )  of   std_logic_vector( 7  downto 0 );

end mytypes;
```
